# Intraoperative dexmedetomidine attenuates norepinephrine levels in patients undergoing transsphenoidal surgery: a randomized, placebo-controlled trial

**DOI:** 10.1186/s12871-020-01025-7

**Published:** 2020-05-02

**Authors:** RyungA Kang, Ji Seon Jeong, Justin Sangwook Ko, Soo-Youn Lee, Jong Hwan Lee, Soo Joo Choi, Sungrok Cha, Jeong Jin Lee

**Affiliations:** 1 Department of Anesthesiology and Pain Medicine, Samsung Medical Center, Sungkyunkwan University School of Medicine, 81 Irwon ro, Gangnam gu, Seoul, 06351 South Korea; 2 Department of Laboratory Medicine and Genetics, Samsung Medical Center, Sungkyunkwan University School of Medicine, Seoul, South Korea

**Keywords:** Dexmedetomidine, Norepinephrine, Stress response, Pituitary

## Abstract

**Background:**

Dexmedetomidine has sympatholytic effects. We investigated whether dexmedetomidine could attenuate stress responses in patients undergoing endoscopic transnasal transseptal transsphenoidal surgery.

**Methods:**

Forty-six patients were randomized to receive a continuous infusion of 0.9% saline (*n* = 23) or dexmedetomidine (n = 23). Immediately after general anesthesia induction, the dexmedetomidine group received a loading dose of 1 mcg/kg dexmedetomidine over 10 min, followed by a maintenance dose of 0.2–0.7 mcg/kg/h and the control group received 0.9% saline at the same volume until 30 min before the end of surgery. Serum levels of epinephrine, norepinephrine, and glucose were assessed before surgery (T1) and the end of drug infusion (T2). The primary outcome was the change in norepinephrine levels between the two time points.

**Results:**

Changes (T2-T1 values) in perioperative serum norepinephrine levels were significantly greater in the dexmedetomidine group than in the control group (median difference, 56.9 pg/dL; 95% confidence interval, 20.7 to 83.8 pg/dL; *P* = 0.002). However, epinephrine level changes did not show significant intergroup differences (*P* = 0.208). Significantly fewer patients in the dexmedetomidine group than in the control group required rescue analgesics at the recovery area (4.3% vs. 30.4%, *P* = 0.047).

**Conclusions:**

Intraoperative dexmedetomidine administration reduced norepinephrine release and rescue analgesic requirement. Dexmedetomidine might be used as an anesthetic adjuvant in patients undergoing transnasal transseptal transsphenoidal surgery.

**Trial registration:**

Clinical Trial Registry of Korea, identifier: KCT0003366; registration date: 21/11/2018; presenting author: Ji Seon Jeong.

## Background

The endoscopic transnasal transseptal transsphenoidal approach (TSA) is commonly used for the excision of pituitary tumors [1]. This surgical approach enables improved visualization of the sellar and parasellar areas, but is associated with an increase in intraoperative stress responses due to intense noxious stimuli at various stages of the surgery, including the insertion of epinephrine-soaked nasal packing, nasal speculum insertion, sphenoidal drilling, and sellar dissection, induce a variety of stress responses [2]. These surgical stimuli induce stress hormone release and sympathetic hyperactivation, resulting in hypertension and tachycardia [3]. To attenuate stress responses during surgery, dexmedetomidine as an anesthetic adjuvant has been used [4, 5].

Dexmedetomidine is a highly selective α2-adrenoceptor agonist, and it has been widely used in the perioperative setting to provide sedation, analgesia, and sympatholysis [6–8]. Recently, dexmedetomidine has become an appealing adjunct during neurosurgical anesthesia because of several properties, including its potential for neuroprotection, minimal impact on neuronal function, stable hemodynamics, opioid- and anesthesia-sparing effects, and minimal respiratory depression [4, 6, 9]. In addition, unlike other sedatives such as propofol, remifentanil, and etomidate, dexmedetomidine has the characteristics of natural sleep-like sedation, in which the patient can still cooperate for a neurologic exam [6]. However, dexmedetomidine may result in hemodynamic compromise including severe bradycardia and hypotension, and thus should be used very cautiously in patients with compromised brain perfusion [6].

In this randomized, placebo-controlled trial, we hypothesized that intraoperative dexmedetomidine administration would reduce intraoperative stress hormone release during TSA surgery. Accordingly, we investigated epinephrine and norepinephrine levels with and without dexmedetomidine administration during TSA surgery.

## Methods

### Study participants

This prospective, randomized, double-blinded, placebo-controlled trial was approved by the Samsung Medical Center Research Ethics Board, Seoul, Korea (SMC 2018–08–022-002), and was prospectively registered with an extension of the Clinical Trial Registry of Korea (http://cris.nih.go.kr; identifier, KCT0003636; principal investigator, Ji Seon Jeong) on November 21, 2018. This study was adhered to CONSORT guideline. One investigator identified eligible patients from the surgeon’s operating list and contacted them the day before surgery to inform them of the study protocol. Written informed consent was obtained from all participants. We enrolled 46 adult patients with American Society of Anesthesiologists Physical Status classification I to III scheduled for elective endoscopic transnasal transseptal TSA surgery for the excision of nonfunctioning pituitary adenoma between January 2019 and April 2019, at Samsung Medical Center, Seoul, Korea. We included only the patients with nonfunctioning pituitary adenoma that were not associated with clinical evidence of hormonal hypersecretion approved by preoperative hormonal tests. We excluded patients with adrenal disorder or pheochromocytoma, as well as those who refused to participate in this study, those with a history of cardiac, renal, or hepatic disease; preexisting atrioventricular block, conduction disorder, or arrhythmia; or allergy to study drugs. We further excluded patients scheduled to undergo planned biopsy of the tumor and revision surgery.

### Randomization and blinding

A member of the Samsung Medical Center who was not otherwise involved in the study performed computer-generated block randomization (www.randomizer.org) at a 1:1 ratio: dexmedetomidine group (*n* = 23) and control group (n = 23). Allocation of patients to each study group was concealed in an opaque envelope that was opened only by the hospital pharmacist who was not involved in either perioperative management or outcome assessment. The hospital pharmacist prepared visually identical study solutions in 50-mL syringes and labeled them using deidentified study code names for double-blinding. The 50-mL syringes contained 2 mL of 200 mcg dexmedetomidine (Dexmedine Inj, Hana Pharmacy, Seoul, Korea) in 48 mL of 0.9% saline to make a total volume of 50 mL (4 mcg/mL) for the dexmedetomidine group, or 50 mL of 0.9% saline for the control group. All enrolled patients, the anesthesiologist performing perioperative management, care givers, every other observer assessing the patient outcomes, and one surgeon who performed all the surgeries were blinded to group allocation until the end of study.

### Interventions and intraoperative management

After applying standard monitoring including electrocardiogram, non-invasive arterial blood pressure measurement, and pulse oximetry, all patients received standardized general anesthesia comprising propofol and remifentanil using syringe pump in target-controlled infusion (TCI) mode (Injectomat TIVA Agilia, Fresinius KABI, France) as a standard of our center. The pharmacokinetic sets used to calculate target effect-site concentrations (Ce) for propofol and remifentanil were Marsh and Minto models, respectively. Ce was set to 3–5 mcg/mL for propofol and 1–3 mcg/mL for remifentanil. During the maintenance of anesthesia, the propofol dose was adjusted to achieve a target bispectral index of 40–60, and the remifentanil dose was adjusted to maintain the mean arterial blood pressure and heart rate within 20% of the preinduction values. Immediately after the induction of general anesthesia, the patients underwent an arterial line cannulation for invasive continuous arterial pressure monitoring and blood sampling, and the study drugs were administered using a syringe pump until 30 min before the end of surgery. The dexmedetomidine group received intravenous dexmedetomidine at a loading dose of 1 mcg/kg over 10 min, followed by a maintenance dose of 0.2–0.7 mcg/kg/h. The maintenance dose of dexmedetomidine was started at a dose of 0.7 mcg/kg/h and was reduced by 0.1 intervals if there was no effect after treatment of hypotension or bradycardia. The control group received intravenous 0.9% saline at the same volume. To treat bradycardia (heart rate < 50 beats per minutes) or hypotension (decreases in mean blood pressure > 20%), the doses of propofol and remifentanil were adjusted first. If it was ineffective, intravenous atropine (0.25–0.5 mg) or ephedrine (2.5–5 mg) was administered for the treatment of bradycardia or hypotension, respectively. After that, we reduced the dose of dexmedetomidine lastly. All patients received 0.1 mg/kg of intravenous hydromorphone before the end of surgery for postoperative analgesia. TCI infusion was maintained until the end of surgery even though the study drug was stopped 30 min before the end of surgery. All patients were extubated after the end of surgery and prior to being transferred to the post-anesthesia care unit (PACU). All surgeries were performed by a single neurosurgeon [1].

### Postoperative management

After the end of surgery, the patients were transferred to the PACU, and stayed there until they met the PACU discharge criteria. Postoperative supplemental analgesia was standardized. Pain severity was measured at rest by using a numerical rating scale (NRS; 11-point scale where 0 = no pain and 10 = worst pain). Patients with NRS scores > 4 were treated using rescue analgesics comprising intravenous ketorolac 30 mg. If this was ineffective after 15 min, intravenous pethidine 25 mg was administered. Postoperative nausea or vomiting was treated using intravenous metoclopramide 10 mg. The level of sedation was assessed during the PACU stay by using the Richmond Agitation-Sedation Scale (RASS) [10], and the duration of sedation was defined as the time from the end of surgery to the time of reaching the score of 0 on the RASS at the PACU. The Glasgow Coma Scale (GCS) was measured at alert and calm state to assess the overall level of consciousness after surgery in PACU. Blinded PACU nurses recorded all PACU data, including opioid consumption, presence or absence of nausea or vomiting, pain scores, and GCS scores.

### Data collection

Blood samples (7 mL) for the measurement of plasma epinephrine and norepinephrine levels were collected through radial arterial cannulation at two predetermined time points: 10 min before surgery (T1), which corresponded to the time immediately after radial arterial cannulation, and the end of drug infusion (i.e., 30 min before the end of surgery) (T2). The collected blood samples were centrifuged, and the plasma and serum were separated and frozen at − 80 °C until analysis. Plasma concentrations of epinephrine and norepinephrine were analyzed by using high-performance liquid chromatography (HPLC) (Agilent 1200 HPLC system, Agilent Technologies, CA, USA) with electrochemical detection by a blinded laboratory investigator. An HPLC kit (Plasma Catecholamines by HPLC, Bio-Rad Laboratories, Hercules, CA, USA) including all reagents, calibrators, controls and column was used. The linear assay range was 10–2000 pg/mL for both epinephrine and norepinephrine. The intra- and inter-day assay precisions were coefficient of variation ≤10% at two concentrations for each analyte. We participated in the proficiency testing provided by the Korean Association of External Quality Assessments Service twice a year. Glucose level was measured using a blood gas/chemistry analysis device (RAPIDLAB1265, Siemens Healthcare Diagnostics Inc., Berlin, Germany) at the same predetermined time intervals (T1 and T2).

Intraoperative hemodynamic variables (mean blood pressure and heart rate) were automatically recorded in the electronic medical records. Intraoperative propofol and remifentanil consumption was also recorded.

### Outcomes

The primary outcome was the change in perioperative serum norepinephrine level. The secondary outcomes included perioperative serum epinephrine and glucose levels, dexmedetomidine-related side effects (hypotension, bradycardia, and sedation), the incidence of postoperative nausea or vomiting, and postoperative pain score measured at discharge from PACU.

### Statistical analysis

The sample size was calculated on the basis of the findings of a previous study by Aho et al. [11] The mean difference (standard deviation [SD]) in serum norepinephrine levels between the baseline and the highest level during surgery between patients who received intramuscular dexmedetomidine and those who received 0.9% saline was 2.2 (1.5) mcg/dL. We expected that, compared to the controls, patients receiving intravenous dexmedetomidine would show a reduction in serum norepinephrine level by at least 50% [12]. We calculated that 21 patients per group were required to detect this degree of difference with a power of 80% and an α = 0.05. Considering a 10% dropout rate, we decided to enroll 46 patients in total.

After determining the normality of data distribution by using the Shapiro-Wilk test, continuous variables were analyzed using the *t* test or Mann-Whitney U test as appropriate. Parametric and non-parametric data were reported as mean ± SDs and median [interquartile ranges], respectively. Categorical variables were analyzed using the chi-square test or Fisher’s exact test. Bonferroni correction was used for multiple comparisons. Data analysis was conducted using IBM SPSS Statistics for Windows/Macintosh, Version 25.0 (IBM Corp., Armonk, NY, USA). For all analyses, a *P*-value < 0.05 was considered significant, and two-sided tests were used.

### Availability of data and materials

The datasets generated and/or analysed during the current study are available from the corresponding author on reasonable request.

## Results

### Study participants

**Fig. 1:**
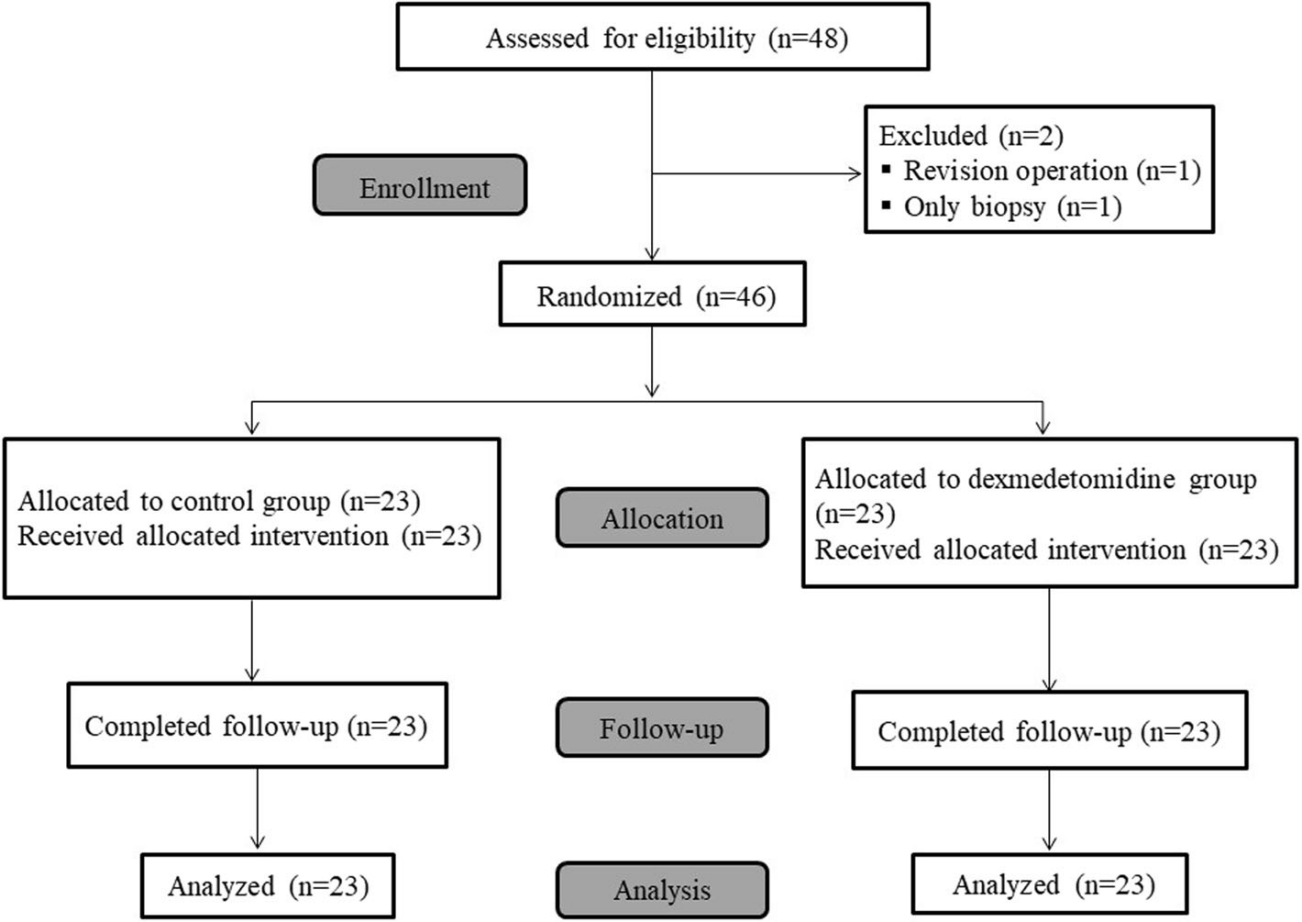
Consolidated Standards of Reporting Trials flow diagram showing patient progress through the study phases

Between January 2019 and April 2019, 48 patients scheduled for TSA surgery for the excision of nonfunctioning pituitary adenoma were assessed for eligibility, and 2 patients were excluded. All enrolled patients were randomly assigned to one of the two study groups (*n* = 23 each) and completed the study (Fig. 1).

All patients underwent their planned surgical procedure and completed the protocol for measurement of the primary outcome. The baseline patient and surgical characteristics were similar between the two groups (Table 1).

**Table 1:** Patients’ characteristics

Parameter	Control (***n*** = 23)	Dexmedetomidine (***n*** = 23)	***P*** value
Age (years)	48 [39–56]	55 [43–62]	0.111
Sex (male/female)	11/12	8/15	0.550
Body mass index (kg/m^2^)	26.0 ± 4.6	25.2 ± 2.8	0.491
ASA physical status (I/II)	18/5	17/6	0.665
Duration of surgery (hours)	2.8 ± 1.6	2.3 ± 1.0	0.262

Values are mean ± standard deviations, median [interquartile ranges], or number. *ASA* American Society of Anesthesiologist

### Outcomes

Changes (T2-T1 values) in perioperative serum norepinephrine levels were significantly greater in the dexmedetomidine group than in the control group (median difference, 56.9 pg/dL; 95% confidence interval [CI], 20.7 to 83.8 pg/dL; P = 0.002). However, changes in perioperative serum epinephrine levels were not significantly affected by the administration of dexmedetomidine (median difference, −12.4 pg/dL; 95% CI, −40.1 to 9.2 pg/dL; *P* = 0.208). Baseline (T1) epinephrine and norepinephrine levels (median [interquartile range]) were not significantly different between the two groups (control vs. dexmedetomidine: epinephrine, 10 [10–22] vs. 11 [10–26] pg/dL, *P* = 0.395; norepinephrine, 153 [125–184] vs. 172 [143–195] pg/dL; *P* = 0.249) (Table 2).

**Table 2:** Perioperative clinical outcomes

Parameters	Control (***n*** = 23)	Dexmedetomidine (***n*** = 23)	***P***^***a***^ value
Stress hormone level at baseline (T1)			
Norepinephrine (pg/mL)	153.3 [125–183.6]	172.3 [142.8–195.2]	0.249
Epinephrine (pg/mL)	10 [10–21.7]	10.6 [10–25.6]	0.395
Glucose (mg/dL)	102 [93–119]	97 [84–108]	0.124
Stress hormone level at the end of infusion (T2)			
Norepinephrine (pg/mL)	102.5 [78.9–143.8]	52.2 [41.2–71.6]	< 0.001
Epinephrine (pg/mL)	35.9 [24.9–43.3]	43.9 [36.1–83.1]	0.066
Glucose (mg/dL)	128 [112–140]	147 [125–161]	0.030
Propofol dose (mg/kg/h)	7.0 [6.3–7.7]	6.8 [6.3–7.2]	0.668
Remifentanil dose (mcg/kg/h)	4.4 [3.4–5.4]	4.5 [3.6–6.3]	0.267
Lowest BIS value during maintenance	40 [40–41]	40 [36–40]	0.088
Highest BIS value during maintenance	50 [47–53]	48 [44–52]	0.136
Intraoperative hypotension, n	6 (26.1%)	9 (39.1%)	0.530
Intraoperative bradycardia, n	1 (4.3%)	9 (39.1%)	0.010
Postoperative nausea or vomiting at PACU, n	4 (17.4%)	1 (4.3%)	0.346
Pain score at PACU (NRS, 0–10)	2 [1–3]	2 [1–2]	0.188
Number of patients requiring rescue analgesics at PACU, n	7 (30.4%)	1 (4.3%)	0.047
GCS score at PACU	15 [15–15]	15 [15–15]	> 0.999
Duration of recovery at PACU (minutes)	60 ± 10	64 ± 9	0.308

Values are mean ± standard deviation, median [interquartile ranges] or number (percentages). ^a^The *P* value for the t-test, Mann-Whitney U test, and the Fisher’s exact test is set at 0.05. *BIS* Bispectral index, *GCS* Glasgow Coma Scale, *PACU* Post-anesthesia care unit

At T2, norepinephrine level was significantly lower in the dexmedetomidine group than in the control group (52 [41–72] vs. 103 [79–144] pg/dL; *P* < 0.001), but epinephrine level was not significantly different between the two groups (control vs. dexmedetomidine: 36 [25–43] vs. 44 [36–83] pg/dL; *P* = 0.066). Glucose level was similar in both groups at T1 (*P* = 0.124), but it was significantly higher in the dexmedetomidine group than in the control group at T2 (control vs. dexmedetomidine: 128 [112–140] vs. 147 [125–161] mg/dL; *P* = 0.03).

**Fig. 2:**
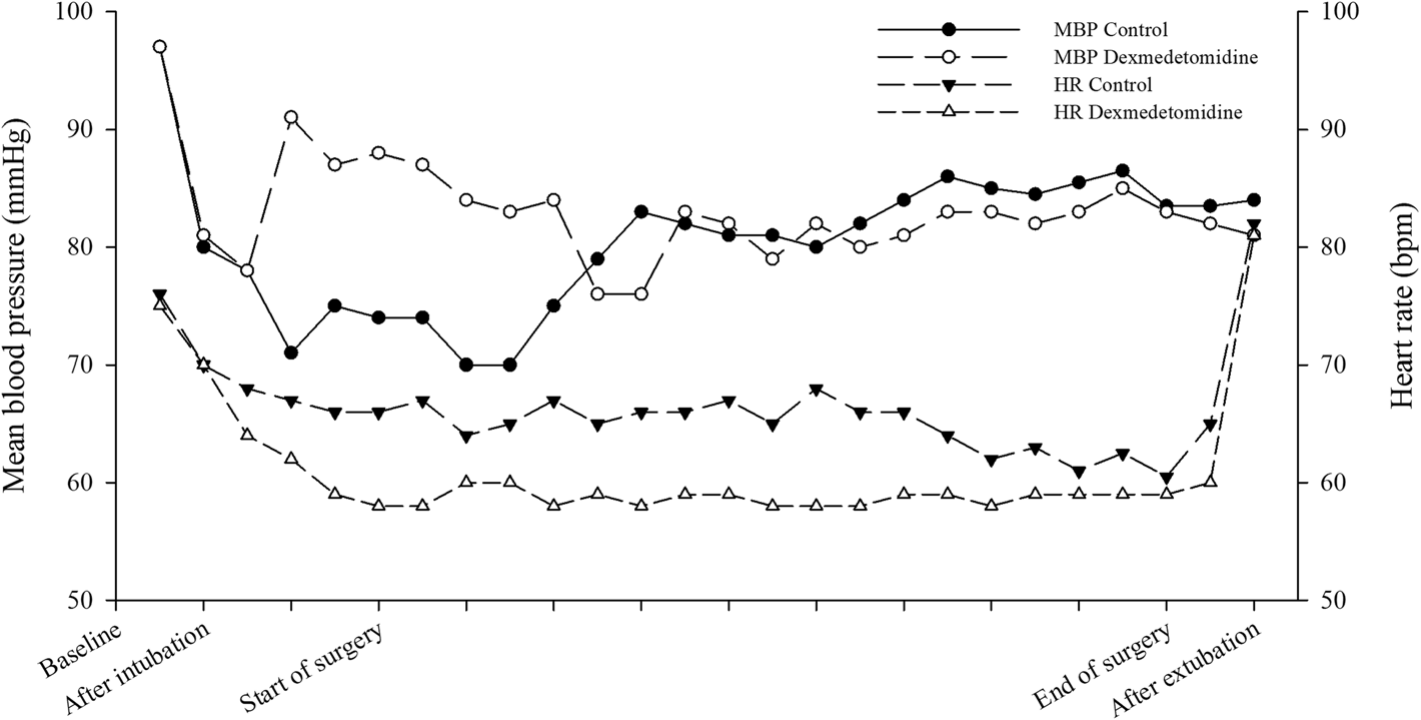
Intraoperative mean blood pressure (MBP) and heart rate (HR) at various times in two groups

Intraoperative mean blood pressure and heart rate are shown in Fig. 2.

Significant hemodynamic instability was not observed in either group. The incidence of intraoperative hypotension was similar between the two groups, but the incidence of intraoperative bradycardia was significantly higher in the dexmedetomidine group than in the control group (Table 2). Postoperative pain score at the recovery area was similar between the two groups (control vs. dexmedetomidine: 2 [1–3] vs. 2 [1–2]; *P* = 0.188). The number of patients requiring rescue analgesics at the PACU was significantly lower in the dexmedetomidine group than in the control group (7 [30.4%] vs. 1 [4.3%] patients; *P* = 0.047). Nevertheless, no significant difference was observed in the length of PACU stay (*P* = 0.308).

## Discussion

In this randomized, placebo-controlled trial, we demonstrated that intraoperative intravenous dexmedetomidine administration attenuates the increase in plasma norepinephrine level during TSA surgery. Patients administered dexmedetomidine showed frequent intraoperative bradycardia but did not show impaired hemodynamic instability and had less requirements for rescue analgesics in the immediate postoperative period.

Surgical stimulus initiates a cascade of stress responses through direct activation of the sympathetic nervous system and increased secretion of pituitary hormones [3, 13]. Attenuation of these stress responses to intense noxious stimuli during surgery improved outcomes by exerting beneficial effects on the postoperative outcomes [14]. Our findings indicated that propofol-remifentanil anesthesia supplemented with dexmedetomidine attenuated surgical stress by inhibiting the secretion of norepinephrine. Dexmedetomidine exerts its sympatholytic effects by activating inhibitory α2-receptors, both in the central nervous system and on peripheral sympathetic nerve endings [3, 11, 12, 14, 15]. However, because plasma norepinephrine passes the blood-brain barrier poorly, attenuated plasma norepinephrine levels do not simultaneously reflect both the central and peripheral sympatholytic effects of dexmedetomidine [16]. Based in this, it can be inferred that plasma norepinephrine was released into the blood from the adrenal medulla in response to sympathetic stimulation separately from the central nervous system. Therefore, attenuated plasma norepinephrine in our study might be due to the peripheral sympatholytic effects of dexmedetomidine. In our study, norepinephrine level was more significantly attenuated in the dexmedetomidine group than in the control group, which showed that dexmedetomidine effectively blunted the stress responses. These findings are consistent with those of a previous study, which showed that dexmedetomidine attenuated catecholamine increase after major spine surgery [12]. However, epinephrine level was unaffected by the administration of dexmedetomidine in our study. The possible reason for this is that dexmedetomidine mainly inhibits release of norepinephrine from presynaptic sympathetic nerve terminals [17]. Another possible reason is that epinephrine-soaked nasal packing may have affected plasma epinephrine levels. Therefore, the true effect of dexmedetomidine on plasma epinephrine level might be masked by epinephrine-soaked nasal packing.

Previous studies have shown that perioperative administration of dexmedetomidine reduced glucose levels that surgical stress produces [14, 18]. However, recent meta-analysis demonstrated that reduced blood glucose levels in dexmedetomidine group were shown in only in abdominal surgery, not in cardiac surgery or other surgery subgroups [3]. In our study, glucose levels were significantly higher in the dexmedetomidine group than in the control group (147 mg/dL vs. 128 mg/dL). The possible reason might be ascribed to the pharmacological properties of the α2-adrenoceptor agonists itself, which can cause hyperglycemia by a mechanism that involves the postsynaptic α2-adrenoceptor stimulation of pancreatic beta cells, which inhibits insulin secretion [19]. Another possible reason might be due to attenuated surgical stress response caused by a dose-related sympatholytic effect of dexmedetomidine based on a study evaluating the effects of dexmedetomidine on glucose levels in children undergoing general anesthesia [20]. Moreover, exogenous epinephrine infusion can cause a transient increase in glucose production and a sustained inhibition of glucose clearance, resulting in hyperglycemia, but is unlikely [21].

The use of intravenous dexmedetomidine during the intraoperative period reduces postoperative opioid consumption [22]. This analgesic-sparing effect was also found in our study. Patients in the dexmedetomidine group had significantly lower requirements of rescue analgesics. Dexmedetomidine has side effects such as hypotension, bradycardia, and oversedation [17]. In our study, typical biphasic hemodynamic effects of dexmedetomidine, including transient hypertension, bradycardia, and hypotension, were explained by the results from the peripheral vasoconstrictive and sympatholytic properties of dexmedetomidine [23]. However, the use of remifentanil with propofol might have obscured the true incidence of hypotension. In addition, a concern regarding the use of dexmedetomidine infusion during surgery is delayed recovery due to the sedative effect of dexmedetomidine. However, in our study, dexmedetomidine administration was discontinued 30 min before the end of surgery, and thus it did not affect the patients’ recovery time [12].

Our study has several limitations. First, we evaluated the effect of dexmedetomidine as an adjuvant under the standard anesthetic regimen of our center (propofol-remifentanil). Therefore, it may be difficult to interpret the true effect of dexmedetomidine owing to study design. In addition, we planned to adjust the maintenance dose of dexmedetomidine, which may impact on the stress response to surgery. However, the loading dose of dexmedetomidine accounts for over 50% of the total dose during surgery. Therefore, we considered the effect of adjustable maintenance dose would be minimal. Second, baseline stress hormone levels were measured immediately after tracheal intubation, which was one of the most important noxious stimuli during surgery. Therefore, these measured values may not reflect the actual reference value. However, since stress hormone levels were measured at the same time point between the two groups, the differences between the two time points could be interpreted as demonstrating the effect of dexmedetomidine. Nevertheless, it would have been better to measure stress hormone levels before the induction of general anesthesia. Third, we measured two types of hormones (epinephrine and norepinephrine) at two time points. The selection of the time points was based on judgment and our experience, but they may not be optimal. The overall effect of dexmedetomidine on surgery could be measured, but the effects of stress intensity, dose, and time of administration could not be measured. Moreover, it would have been better to measure cortisol, a more suitable marker for stress response to evaluate stress response [3]. Further study is needed for the serial measurement of the effects of dexmedetomidine at various stages of surgery, as is the incorporation of other stress responses markers, such as cortisol, glucagon, plasma interleukin-6 [19], to establish more accurate responses to the administration of dexmedetomidine.

## Conclusions

In conclusion, intraoperative dexmedetomidine administration reduced norepinephrine release and reduced rescue analgesic requirements. Dexmedetomidine can be used as an anesthetic adjuvant in patients undergoing TSA surgery.

## Data Availability

The datasets generated and/or analysed during the current study are available from the corresponding author on reasonable request.

## Abbreviations

GCS: Glasgow Coma Scale
PACU: Post-anesthesia care unit
TSA: Transsphenoidal approach
NRS: Numerical rating scale
RASS: Richmond Agitation-Sedation Scale

## References

[1] Hong SD, Nam DH, Kong DS, Kim HY, Chung SK, Dhong HJ (2016). Endoscopic modified transseptal transsphenoidal approach for maximal preservation of sinonasal quality of life and olfaction. World Neurosurg.

[2] Jan S, Ali Z, Nisar Y, Naqash IA, Zahoor SA, Langoo SA, Azhar K (2017). A comparison of dexmedetomidine and clonidine in attenuating the hemodynamic responses at various surgical stages in patients undergoing elective transnasal transsphenoidal resection of pituitary tumors. Anesth Essays Res.

[3] Wang K, Wu M, Xu J, Wu C, Zhang B, Wang G, Ma D (2019). Effects of dexmedetomidine on perioperative stress, inflammation, and immune function: systematic review and meta-analysis. Br J Anaesth.

[4] Kadarapura N, Gopalakrishna PKD, Chatterjee N, Hariharan V. Easwer, and Arimanickam Ganesamoorthi: Dexmedetomidine as an anesthetic adjuvant in patients undergoing transsphenoidal resection of pituitary tumor. J Neurosurg Anesthesiol. 2015;27:209–15. 10.1097/ANA.0000000000000144 25493927

[5] Gupta D, Srivastava S, Dubey RK, Prakash PS, Singh PK, Singh U (2011). Comparative evaluation of atenolol and clonidine premedication on cardiovascular response to nasal speculum insertion during trans-sphenoid surgery for resection of pituitary adenoma: a prospective, randomised, double-blind, controlled study. Indian J Anaesth.

[6] Lin N, Vutskits L, Bebawy JF, Gelb AW. Perspectives on dexmedetomidine use for neurosurgical patients. J Neurosurg Anesthesiol. 2019;31(4):366–77. 10.1097/ANA.0000000000000554 30363004

[7] Hall JE, Uhrich TD, Barney JA, Arain SR, Ebert TJ (2000). Sedative, amnestic, and analgesic properties of small-dose dexmedetomidine infusions. Anesth Analg.

[8] Lee S (2019). Dexmedetomidine: present and future directions. Korean J Anesthesiol.

[9] Darmawikarta D, Sourour M, Couban R, Kamath S, Reddy KK, Shanthanna H (2019). Opioid-free analgesia for supratentorial craniotomies: a systematic review. Can J Neurol Sci.

[10] Sessler CN, Gosnell MS, Grap MJ, Brophy GM, O’Neal PV, Keane KA, Tesoro EP, Elswick RK (2002). The Richmond agitation-sedation scale: validity and reliability in adult intensive care unit patients. Am J Respir Crit Care Med.

[11] Aho M, Scheinin M, Lehtinen AM, Erkola O, Vuorinen J, Korttila K (1992). Intramuscularly administered dexmedetomidine attenuates hemodynamic and stress hormone responses to gynecologic laparoscopy. Anesth Analg.

[12] Kim MH, Lee KY, Bae SJ, Jo M, Cho JS (2019). Intraoperative dexmedetomidine attenuates stress responses in patients undergoing major spine surgery. Minerva Anestesiol.

[13] Desborough JP (2000). The stress response to trauma and surgery. Br J Anaesth.

[14] Uyar AS, Yagmurdur H, Fidan Y, Topkaya C, Basar H (2008). Dexmedetomidine attenuates the hemodynamic and neuroendocrinal responses to skull-pin head-holder application during craniotomy. J Neurosurg Anesthesiol.

[15] Keniya VM, Ladi S, Naphade R (2011). Dexmedetomidine attenuates sympathoadrenal response to tracheal intubation and reduces perioperative anaesthetic requirement. Indian J Anaesth.

[16] Patrick A, Vdq S, Foti A, Curzon G (1986). Effects of clonidine on central and peripheral nerve tone in primary hypertension. Hypertension.

[17] Ralph Gertler HCB, Mitchell DH, Silvius EN (2001). Dexmedetomidine: a novel sedative-analgesic agent. BUMC Proc.

[18] Harsoor SS, Rani DD, Lathashree S, Nethra SS, Sudheesh K (2014). Effect of intraoperative dexmedetomidine infusion on sevoflurane requirement and blood glucose levels during entropy-guided general anesthesia. J Anaesthesiol Clin Pharmacol.

[19] AB RMV, Hall GM, Grounds RM (2001). Effects of dexmedetomidine on adrenocortical function, and the cardiovascular, endocrine and inflammatory responses in postoperative patients needing sedation in the intensive care unit. Br J Anaesth.

[20] Gorges M, Poznikoff AK, West NC, Brodie SM, Brant RF, Whyte SD (2019). Effects of dexmedetomidine on blood glucose and serum potassium levels in children undergoing general anesthesia: a secondary analysis of safety endpoints during a randomized controlled trial. Anesth Analg.

[21] Vranic M, Gauthier C, Bilinski D, Wasserman D, El Tayeb K, Hetenyi G, Lickley HL (1984). Catecholamine responses and their interactions with other glucoregulatory hormones. Am J Phys.

[22] Kang R, Jeong JS, Yoo JC, Lee JH, Choi SJ, Gwak MS, Hahm TS, Huh J, Ko JS (2018). Effective dose of intravenous Dexmedetomidine to prolong the analgesic duration of Interscalene brachial plexus block: a single-center, prospective, double-blind, randomized controlled trial. Reg Anesth Pain Med.

[23] Weerink MA, Struys MM, Hannivoort LN, Barends CR, Absalom AR, Colin P. Clinical pharmacokinetics and pharmacodynamics of dexmedetomidine. Clin Pharmacokinet. 2017;56(8):893–913. 10.1007/s40262-017-0507-7 PMC5511603 28105598

